# Mid-Term Results of Peripheral Cannulation After Robotic Cardiac Surgery

**DOI:** 10.21470/1678-9741-2018-0061

**Published:** 2018

**Authors:** Onur Sen, Unal Aydin, Ersin Kadirogullari, Muhammed Bayram, Mehmet Karacalilar, Erhan Kutluk, Burak Onan

**Affiliations:** 1 Department of Cardiovascular Surgery, İstanbul Mehmet Akif Ersoy Thoracic and Cardiovascular Surgery Training and Research Hospital, Istanbul, Turkey.

**Keywords:** Robotic Surgical Procedures, Surgery, Computer-Assisted, Femoral Artery, Ultrasonography, Doppler, Vascular Diseases

## Abstract

**Introduction:**

Various surgical procedures for minimally invasive cardiac surgery have been described in recent decades as alternatives to median sternotomy. Cardiopulmonary bypass via femoral arterial and venous cannulation is the foundation of these procedures. In this study, we evaluated the mid-term outcomes of femoral cannulation performed with U-suture technique in patients undergoing robotic heart surgery.

**Methods:**

A total of 216 patients underwent robotic-assisted cardiac surgery between January 2013 and April 2017. Cardiopulmonary bypass was performed via femoral artery, jugular, and femoral vein cannulation, and a Chitwood clamp was used for aortic occlusion. A total of 192 patients attended the outpatient follow-up, and femoral arterial and venous flow pattern was examined using Doppler ultrasound (DUS) in 145 patients.

**Results:**

Hospital mortality occured in 4 of the 216 (1.85%) cases, but there was no late mortality in this patient group. Postoperatively, seroma (n=9, 4.69%) and cannulation site infection (n=3, 1.56%) were managed with outpatient treatment. DUS in 145 patients revealed triphasic flow pattern in the common femoral arteries in all patients except for 2 (1.38%). These patients were determined to have asymptomatic arterial stenosis. Chronic recanalized thrombus in the common femoral vein was also detected in 2 (1.38%) patients.

**Conclusion:**

Femoral artery cannulation with the U-suture technique can be successfully performed in robotic-assisted cardiac surgery, with good mid-term results.

**Table:** 

Abbreviations, acronyms & symbols	
ASD	= Atrial septal defect
BMI	= Body mass index
CPB	= Cardiopulmonary bypass
DUS	= Doppler ultrasound
PTFE	= Polytetrafluoroethylene
TEE	= Transesophageal echocardiography

## INTRODUCTION

Median sternotomy with cardiopulmonary bypass (CPB) using conventional methods of ascending aortic and right atrial or bicaval cannulation has been the standard approach to the majority of cardiac surgical procedures. However, minimally invasive techniques involving surgical exploration and cannulation without median sternotomy have been described in recent years^[1-5]^. In minimally invasive procedures, CPB requires cannulation of the right internal jugular vein and right femoral artery and vein. However, complications associated with peripheral cannulation are sometimes observed^[1-3]^. We have been performing peripheral cannulation methods such as the U-suture technique in our unit since 2012. In this study, we aimed to evaluate the clinical results and vascular flow patterns after peripheral cannulation for robotic cardiac surgery.

## METHODS

A total of 216 consecutive robotic valvular and atrial septal defect (ASD) operations were performed between January 2013 and April 2017. The mean follow-up time was 25.7 months (range 2-44 months). Patients who were not eligible for robotic surgery and therefore could not undergo minimally invasive surgery were excluded. This group included patients with severe coronary artery disease, obesity (body mass index [BMI] > 30), aortic valve insufficiency, lung adhesions, chest wall deformities, or iliofemoral artery disease. All patients underwent detailed preoperative examination, including computerized tomography, as well as transesophageal echocardiography (TEE) to assess surgical risk. Excluding patients who died or were lost to follow-up, a total of 192 patients attended outpatient follow-up, and femoral arterial and venous flow pattern was evaluated with Doppler ultrasound (DUS) in 145 patients. The patients' demographic data and risk factors are shown in Table 1. Most of the procedures performed were valve surgeries (Table 2).

**Table 1:** Demographic parameters of patients who underwent robotic cardiac surgery with U-suture peripheral cannulation.

Patient characteristics	
Patients, n	216
Patient age, years	44±7
Male/female, n	102/114
Hypertension, n	46
Smoking, n	124
Obesity, n	0
LVEF, %	53±7.1

LVEF=left ventricular ejection fraction; n=number

**Table 2:** Types of robotic cardiac operations in the study.

Type of operation	Patients (n)
MVR	32
ASD *secundum*	51
MVrp	57
MVR + TVrp	27
MVrp + TVrp	24
ASD + TVrp	25

ASD=atrial septal defect; MVR=mitral valve replacement; MVrp=mitral valve repair; TVrp=tricuspid valve repair

Data are given as mean (max-min) or percentage. The mean is calculated by dividing the total of data by number of data. Percentage is meaning the ratio of data as a fraction of 100.

### Surgical Procedure

**Fig. 1:**
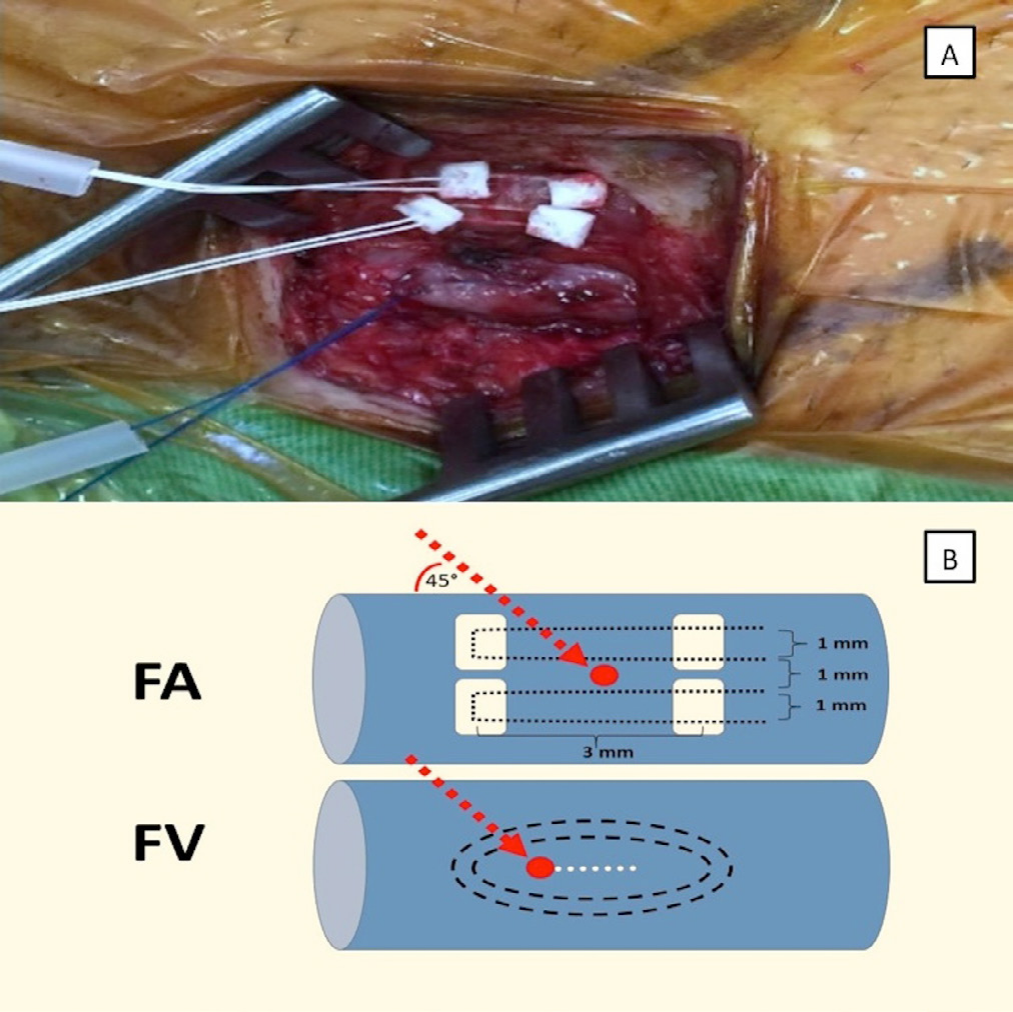
A) Placement of double pledgeted polytetrafluoroethylene sutures in the femoral artery and Prolene sutures in the common femoral vein. B) Double-pledgeted sutures are placed horizontally through the adventitial layer of the femoral artery (FA). The red dot shows the puncture site between the two-layered horizontal sutures. Note the lengths and distances between the sutures. Double purse-string sutures were placed in the anterior surface of the common femoral vein (FV). The red dot shows the puncture site and the dotted white line shows the 3 mm incision made before insertion of the venous cannula.

**Fig. 2:**
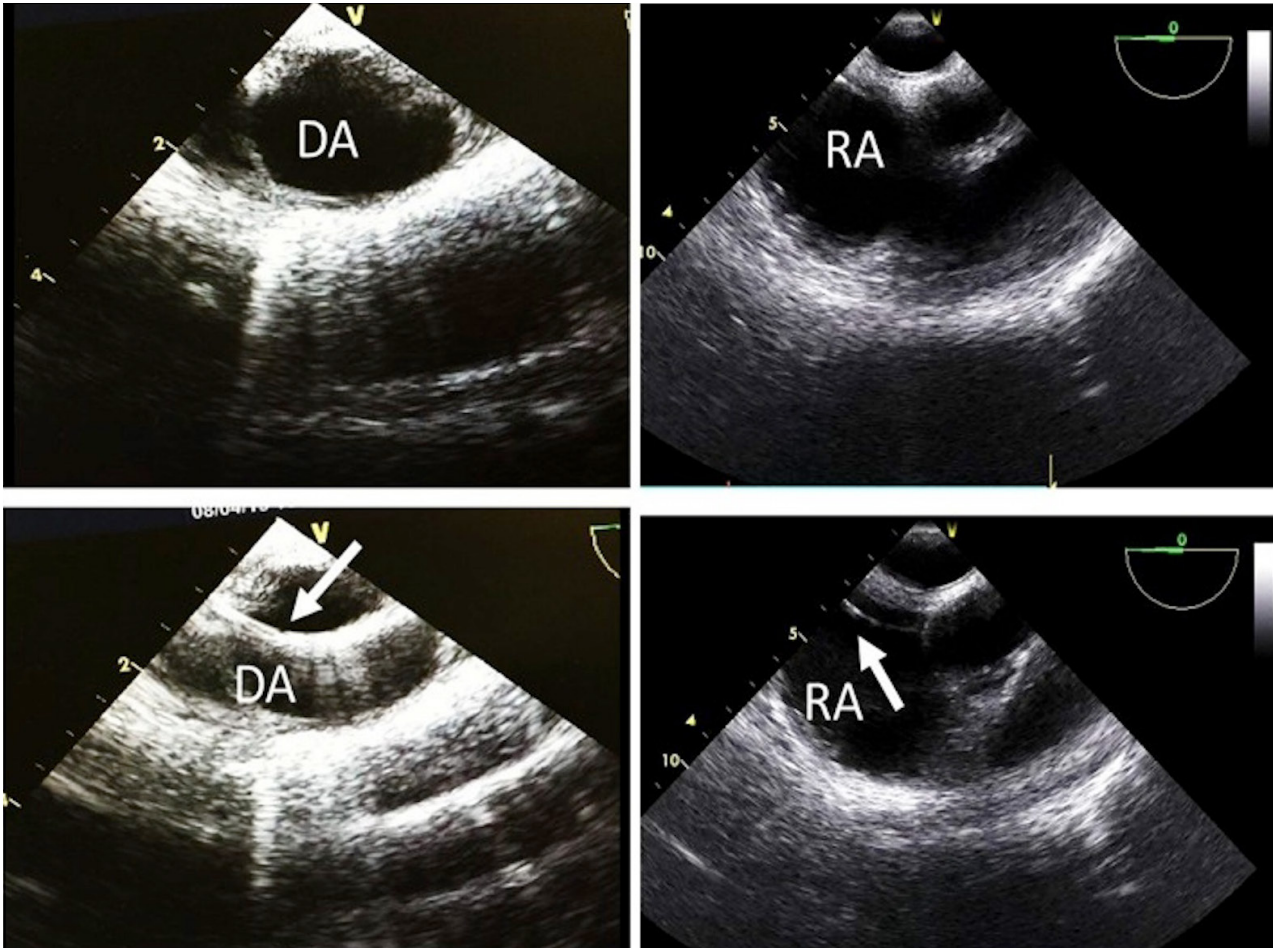
Intraoperative transesophageal echocardiography guidance images during peripheral cannulation. In the lower left panel, the guidewire (arrow) is visible in the descending aorta (DA). In the lower right panel, the guidewire (arrow) is seen in the right atrium (RA).

All procedures were performed under general anesthesia with single-lumen intubation. Superior vena cava cannulation was performed via percutaneous access to the right internal jugular vein with 17-F arterial cannula (DLP, Inc., Grand Rapids, MI, USA) following an injection of 100 IU/kg of heparin. External defibrillation pads were placed, and patients were positioned supine with right chest elevated by 30 degrees. A soft tissue retractor (Alexis Retractor, Applied Medical, CA, USA) was used to expose the surgical area without rib intervention. The right femoral artery and vein were surgically accessed through a groin incision and another 300 IU/kg of heparin was administered before cannulation. Two 5-0 polypropylene purse-string sutures were placed in the femoral vein and a 24-29-F venous cannula (DLP Inc., Grand Rapids, MI, USA) was inserted through the purse-string sutures. Under TEE guidance, the tip of the venous cannula was placed at the junction of the inferior vena cava and right atrium over a flexible J-wire. Arterial cannulation was performed using the U-suture technique. The common femoral artery was explored, and 5-0 propylene or 3-0 polytetrafluoroethylene (PTFE) pledgeted sutures were placed on the left and right sides of the femoral artery, leaving an area of approximately 2-5 mm in length and width between the sutures (Figures 1A and B). The 18-20-F arterial cannula (DLP Inc., Grand Rapids, MI, USA) was inserted into the common femoral artery between the two U-sutures using the Seldinger technique. TEE guidance was routinely used to follow the guidewire during cannulation in order to avoid cannula malposition and vascular traumas such as dissection and rupture. Furthermore, TEE was also used to evaluate valve dynamics and detect evacuation of air (Figure 2). Upon completion of the cannulation procedure, CPB was initiated and lung ventilation was stopped. The ascending aorta was occluded using a Chitwood transthoracic aortic cross-clamp (Aesculap Inc., Corporate Parkway Center Valley, PA, USA). The Chitwood clamp was placed through the third intercostal space along the right anterior axillary line, then cold antegrade crystalloid Bretschneider's cardioplegia was administered to the aortic root to arrest the heart.

**Fig. 3:**
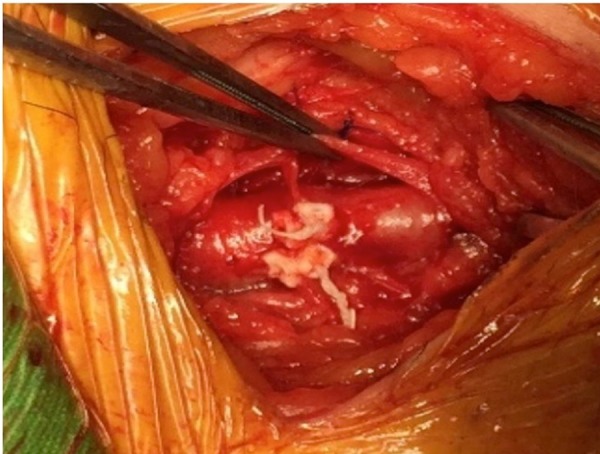
Appearance of the femoral artery and vein after tying the sutures.

After completing the procedure and terminating CPB, the femoral venous and arterial cannulae were removed and the sutures were securely knotted (Figure 3). Arterial cannulation and decannulation were uneventful in most patients. At the end of the operation, the jugular vein cannula was removed and moderate pressure was applied to the incision site for 15 minutes.

### Doppler Ultrasound Examination 

The DUS evaluation of the venous and arterial flow rate and pattern was performed using a Siemens SONOLINE Anteres ultrasound system (Germany) with a 7-3 MHz probe. Peak velocity increases in areas of stenosis in proportion to the degree of narrowing. It was observed that, at the proximal site of the cannulation segment, significant increase at the peak systolic velocity corresponded to lesions of 50% or more narrowing in the luminal diameter of the artery. At the distal segment of the cannulation site, the peak systolic velocity was measured as equal or lower than the proximal stenotic segment measurements. Color Doppler imaging was used to detect turbulent flow. The venous caliber and the presence of turbulent flow were evaluated in venous examination.

## RESULTS 

A total of 216 patients underwent robotic-assisted cardiac surgery during the study period. Of these patients, 4 (1.85%) died in the hospital and 20 (9.26%) were lost to follow-up, leaving 192 patients under outpatient follow-up. The mean follow-up was 25.7 months (range 2-44 months) and the mean postoperative hospital stay was 6 days (range 5-9 days). There was no late mortality. Postoperatively, cannulation site infection occurred in 3 (1.56%) patients and seroma in 9 (4.69%), all of whom were successfully treated with outpatient care (Table 3).

**Table 3:** Surgical parameters and postoperative mortality and peripheral vascular parameters and complications in patients who underwent robotic cardiac surgery with femoral cannulation using the U-suture technique.

Operative and postoperative patient characteristics	
CPB time, min	132±29.6
Cross-clamp time, min	89±17.1
Mortality, n (%)	4/216 (1.85)
Wound infections, n (%)	3/192 (1.56)
Seroma, n (%)	9/192 (4.69)
Mean CFA flow rate, cm/s	164.28
Mean CFV diameter, mm	8.2
CFA stenosis, n (%)	2/145 (1.38)
CFV thrombosis, n (%)	2/145 (1.38)

CFA=common femoral artery; CFV=common femoral vein; CPB=cardiopulmonary bypass

In the 145 patients examined with DUS for femoral arterial and venous stenosis, flow rate was calculated as 70-178 cm/s (mean 124.12 cm/s) and common femoral vein diameter was 5.9-12.8 mm (mean 8.2 mm). Flow pattern in the common femoral arteries was triphasic in all patients except for 2 (1.38%). In these 2 patients, DUS of the common femoral artery revealed 60% narrowing of the lumen, monophasic flow pattern, and subnormal flow rates (32 cm/s and 35 cm/s). These patients remain under outpatient follow-up for arterial stenosis with no complications or complaints to date. Chronic recanalized thrombosis in the common femoral vein was detected in 2 (1.38%). DUS showed luminal narrowing and thickening of the common femoral vein wall. No venous turbulent flow was detected.

## DISCUSSION

In this study, we retrospectively evaluated complications related to peripheral cannulation in patients who underwent robotic cardiac surgery. Our aim was to determine whether the technique we used led to any differences in postoperative complications compared to other peripheral cannulation techniques in robotic cardiac surgery.

Robotic mitral valve surgery is among the most commonly performed robotic cardiac procedures in the last decade. The benefits include smaller, less invasive incisions, less incisional pain, shorter hospital stay, better cosmesis, quicker return to usual life activities, and reduced blood loss and need for transfusion^[6]^. However, femoral artery and vein cannulation is required for CBP and may result in vascular injury. In our practice, we use femoral artery and vein cannulation for CPB and Chitwood clamping of the ascending aorta, as described by Chitwood et al.^[7]^, to avoid complications and reduce the cost of the surgery. In general, operative times and results were similar to those of conventional surgical techniques^[6]^.

It has been reported that prolonged peripheral artery cannulation in patients undergoing minimally invasive operations can cause limb ischemia and potentially lead to ischemic complications^[8,9]^. In our series, the CPB and ischemic times did not exceed the expected limits. We observed no ischemic complications in the cannulated extremities that required thrombectomy and/or fasciotomy in the early postoperative period.

Muhs et al.^[10]^ investigated arterial injuries related to femoral artery cannulation in 739 consecutive patients who underwent minimally invasive cardiac surgery. They reported postoperative claudication in 4 patients, 3 of whom had iliofemoral arterial occlusion or localized iliofemoral dissection and were treated with an iliofemoral bypass; another patient had localized femoral artery stenosis which was treated by angioplasty. In our series of 192 patients, none complained of claudication. We believe this is attributable to the U-suture technique, which does not require femoral arteriotomy or clamping.

All of our patients were followed in the outpatient clinic due to problems related to femoral artery and venous cannulation, and patients with possible flow abnormalities were evaluated by DUS. Although stenosis was detected in the common femoral artery in two of our patients, they have shown no symptoms and have not required treatment. The two patients with chronic recanalized thrombosis in the common femoral vein are also asymptomatic. Overall, vascular complications were minor and infrequent in our series.

In routine practice, the femoral transverse arteriotomy technique is commonly used in various departments^[3,10]^. Our technique provides optimal arterial lumen diameter and reduces the risk of arterial thrombosis and femoral hematoma. However, comparison of these two techniques was not possible in the present study, since we only utilize the method described above in our department. Nevertheless, this study demonstrates that the U-suture technique has low complication rates when applied in the context of robotic cardiac surgery.

### Limitation 

A limitation of this study is that different cannulation techniques cannot be directly compared in our center. Furthermore, the mean follow-up time was not very long. Because this is a relatively new cardiac center, the single-center and retrospective design of this study is a limitation.

## CONCLUSION

In conclusion, femoral artery cannulation for robotic heart surgery with U-suture technique can be successfully performed with good mid-term peripheral vascular outcomes.

**Table:** 

Authors' roles & responsibilities	
OS	Conception and study design; analysis and/or data interpretation; statistical analysis; final manuscript approval
UA	Conception and study design; analysis and/or data interpretation; statistical analysis; final manuscript approval
EK	Conception and study design; analysis and/or data interpretation; statistical analysis; final manuscript approval
MB	Conception and study design; analysis and/or data interpretation; statistical analysis; final manuscript approval
MK	Conception and study design; analysis and/or data interpretation; statistical analysis; final manuscript approval
EK	Conception and study design; analysis and/or data interpretation; statistical analysis; final manuscript approval
BO	Execution of operations and/or trials; manuscript writing or critical review of its content; final manuscript approval
